# Supplementary figures and images for: Transcriptome Analysis Reveals Candidate Genes Involved in Gibberellin-Induced Fruit Development in Rosa roxburghii
Source: Plants (Basel). 2023 Sep 28;12(19):3425. doi: 10.3390/plants12193425 (PMC10575181; doi:10.3390/plants12193425)

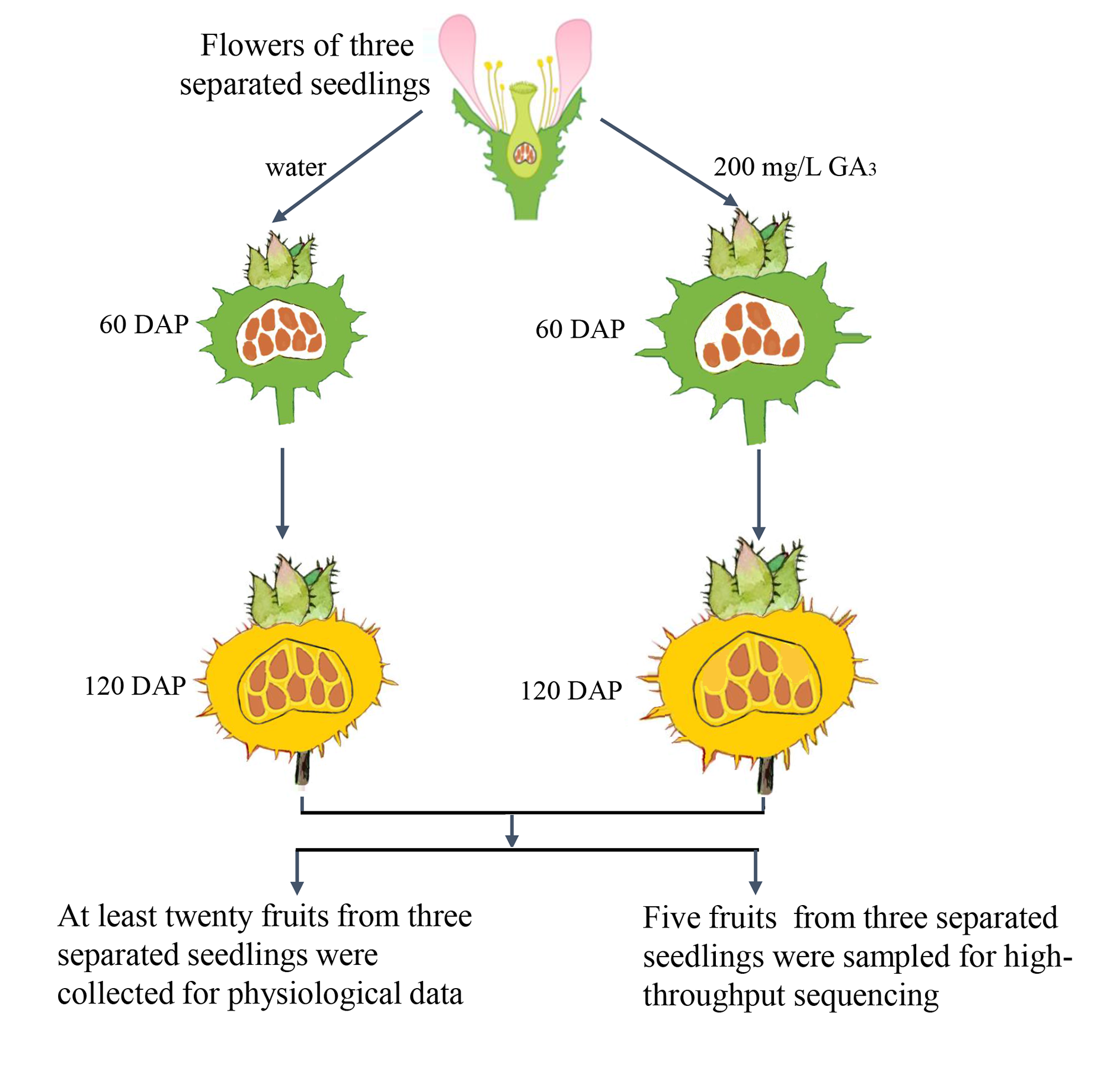

Supplement: Supplementary file 1 [file plants-12-03425-s001.zip › Figure S1.png]
